# Rosai-Dorfman Disease Presenting as a Bone-Destructive Mass in the Maxillary Sinus With Orbital Extension: A Diagnostic and Therapeutic Challenge

**DOI:** 10.7759/cureus.85870

**Published:** 2025-06-12

**Authors:** Miki Junicho, Akira Nakazato, Hirohiko Tachino, Yuka Morita

**Affiliations:** 1 Department of Otorhinolaryngology, Head and Neck Surgery, Faculty of Medicine, University of Toyama, Toyama, JPN

**Keywords:** bone destruction, maxillary sinus tumor, non-langerhans cell histiocytosis, rosai-dorfman disease, vertical diplopia

## Abstract

Rosai-Dorfman disease (RDD) is a rare histiocytic disorder characterized by massive lymphadenopathy and, in some cases, extranodal involvement, resulting in significant functional impairment. Maxillary sinus lesions with orbital extension are extremely rare and may mimic malignancy. We report the case of a 76-year-old man who presented with left lower eyelid swelling and diplopia. Computed tomography revealed a mass centered in the left maxillary sinus, extending into the orbit and buccal subcutaneous tissue, accompanied by osteolytic changes. Serologic testing, including soluble interleukin-2 receptor (sIL-2R) and immunoglobulin G4 (IgG4) level, was unremarkable. An initial nasal biopsy suggested chronic inflammation; however, surgical excision was performed due to concerns about malignancy. Histopathology revealed S-100- and CD68-positive histiocytes exhibiting emperipolesis, confirming a diagnosis of RDD. The patient was treated with oral corticosteroids (prednisolone 1 mg/kg/day), resulting in rapid symptom resolution without recurrence over an 18-month follow-up period. This case highlights the importance of including RDD in the differential diagnosis of bone-destructive maxillary sinus lesions. Although RDD is not a malignant tumor, it can cause significant functional impairment. Corticosteroids appear to be an effective treatment option. Early diagnosis and appropriate steroid therapy may lead to excellent outcomes while avoiding overtreatment.

## Introduction

Rosai-Dorfman disease (RDD) is a rare non-Langerhans cell histiocytosis, typically presenting with massive, painless cervical lymphadenopathy. Extranodal involvement is observed in 40%-50% of cases and may occur in sites such as the skin, orbit, nasal cavity, and paranasal sinuses [1]. Isolated sinonasal involvement is particularly rare and can present with aggressive features, including bone destruction, mimicking malignancies. Here, we present a case of RDD centered in the maxillary sinus with orbital extension, without cervical lymphadenopathy, initially suspected to be malignant due to its radiographic and clinical features.

## Case presentation

A 76-year-old man was referred to our Otorhinolaryngology Department following an incidental finding on a head computed tomography (CT) scan, performed by his primary physician. The scan revealed a soft tissue-dense lesion in the left maxillary sinus, extending into the orbit and buccal subcutaneous tissue, associated with osteolytic changes of the anterior and superior sinus walls.

Clinically, the patient reported no nasal symptoms but complained of swelling in the left lower eyelid and vertical diplopia. Ophthalmological evaluation confirmed restricted upward gaze in the left eye, without vision impairment. No cervical lymphadenopathy was noted, and endoscopic examination of the nasal cavity revealed no abnormalities.

**Figure 1:**
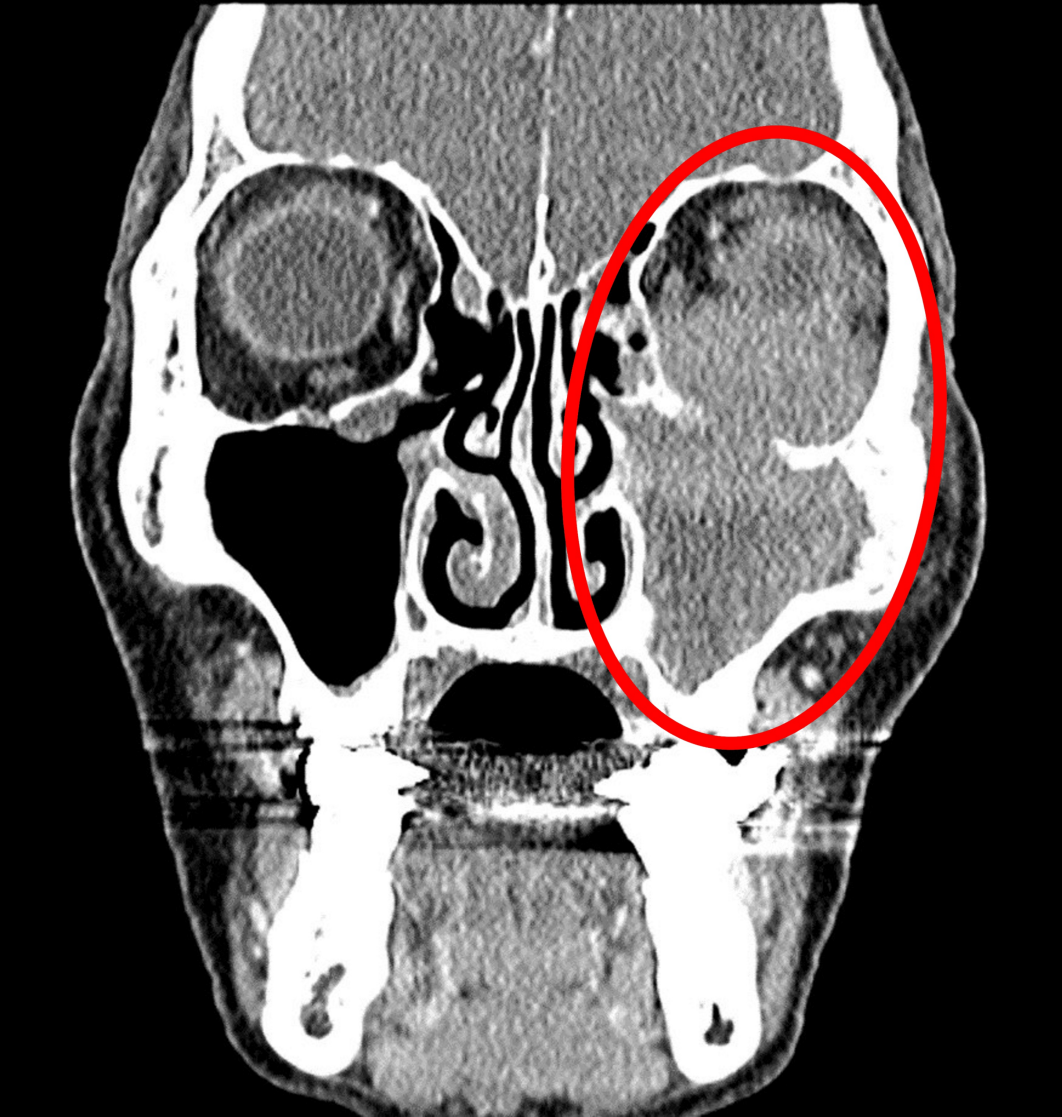
Contrast-enhanced CT image Occupying lesion centered on the left maxillary sinus, with uniform mild contrast in the left orbit and buccal subcutis (circle area). CT, computed tomography

**Figure 2:**
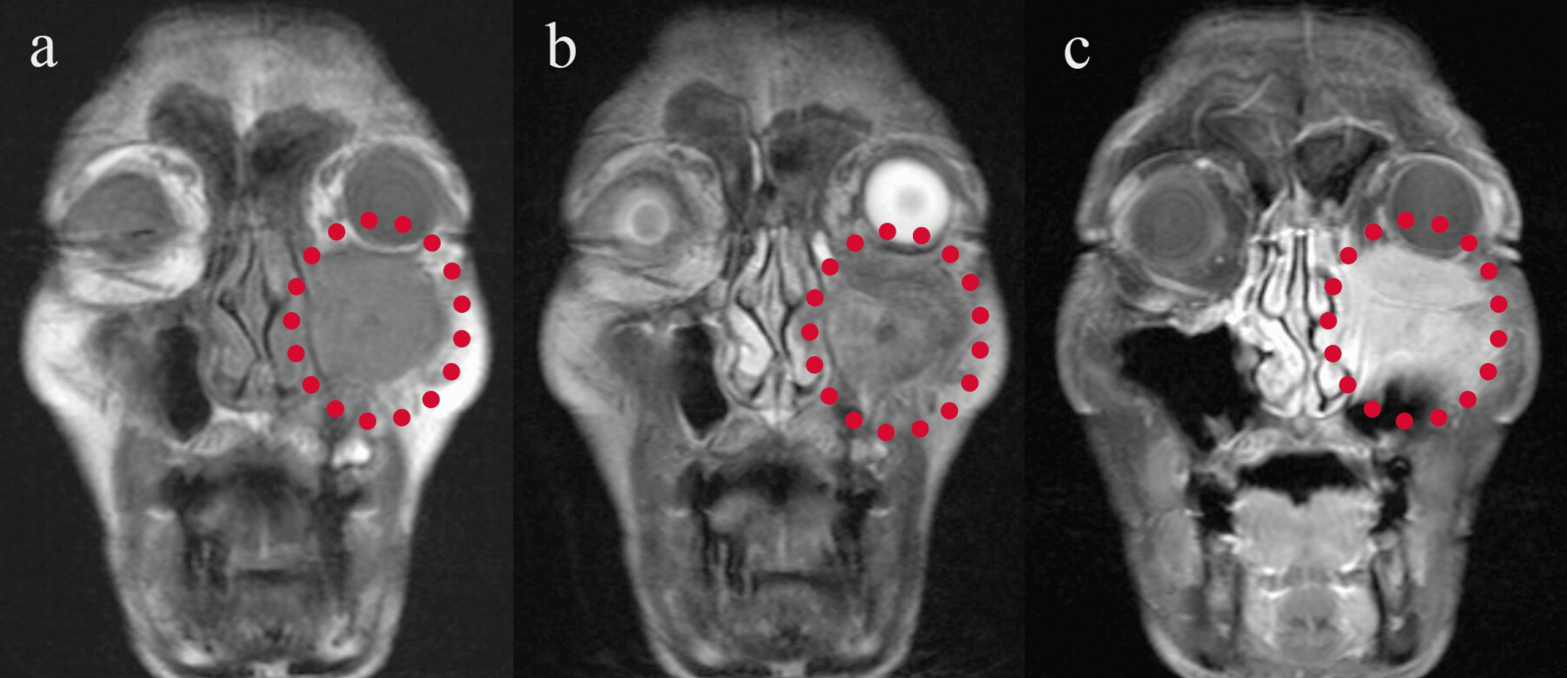
MRI images An abnormal lesion (circle area), with low signal on T1 (a) and high signal on T2 (b), is seen, and the contrast effect extends into the orbit on contrast T1 (c). MRI, magnetic resonance imaging

Laboratory investigations showed no elevation in inflammatory markers. Serum soluble interleukin-2 receptor (sIL-2R) and immunoglobulin G4 (IgG4) levels were within normal limits (sIL-2R: 304.1 U/mL, IgG4: 33.0 mg/dL; Table 1). Contrast-enhanced CT revealed a mildly enhancing mass extending from the left maxillary sinus into the orbit and buccal region (Figure 1). Magnetic resonance imaging (MRI) showed the lesion to be hypointense on T1, hyperintense on T2, and enhancing with gadolinium, with unclear borders between the mass and the inferior rectus muscle (Figure 2).

**Table 1:** Laboratory investigations Laboratory investigations show no elevation in inflammatory markers.

Test	Patient Value	Normal Reference Values
White blood cell count	5430 /μL	3300 ~ 8600 /µL
Red blood cell count	389 × 10^4 /µL	435 ~ 555 × 10^4 /µL
Hemoglobin	11.0 g/dL	13.7 ~ 16.8 g/dL
Platelet count	31.8 × 10^4 /µL	15.8 ~ 34.8 × 10^4 /µL
C-reactive protein	0.12 mg/dL	0.00 ~ 0.14 mg/dL
Soluble interleukin-2 receptor	304.1 U/mL	157.0 ~ 474.0 U/mL
Immunoglobulin G4 (IgG4)	33.0 mg/dL	11.0 ~ 121.0 mg/dL
Squamous cell carcinoma antigen	1.0 ng/mL	0.0 ~ 2.5 ng/mL

**Figure 3:**
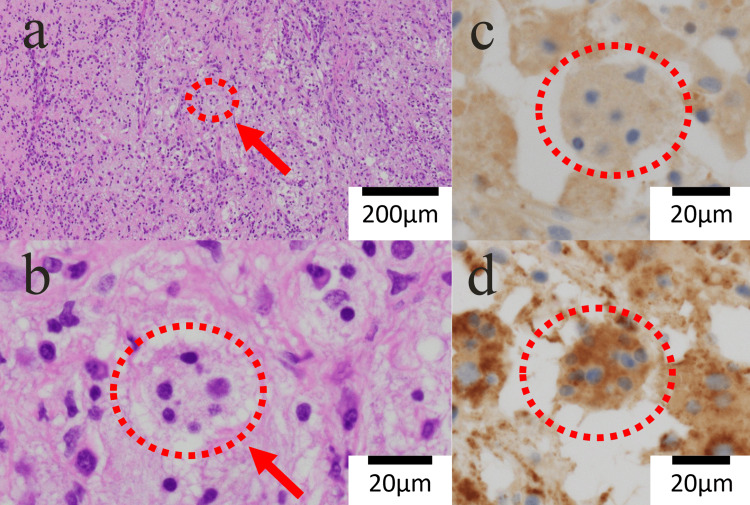
Immunohistochemical staining images (a) HE (low magnification), (b) HE (high magnification), (c) S-100, (d) CD68 HE-stained (a & b): A high degree of inflammatory cell infiltration - mainly lymphocytes and plasma cells - and emperipolesis (circle & arrow) by histiocytes are observed. S-100-stained (c) and CD68-stained (d): The histiocytoid cells (circle) showed positive immunostaining for S-100 protein and CD68 protein.

A nasal biopsy under local anesthesia revealed only nonspecific inflammatory changes. Due to persistent suspicion of malignancy, the patient underwent endoscopic tumor excision with a CT navigation system under general anesthesia. Intraoperatively, the lesion appeared as thickened mucosal tissue with inflammatory characteristics. Histopathology revealed large histiocytes showing emperipolesis, accompanied by a dense infiltrate of lymphocytes and plasma cells. Immunohistochemistry was positive for S-100 protein and CD68, and negative for CD1a (Figure 3), confirming the diagnosis of RDD.

**Figure 4:**
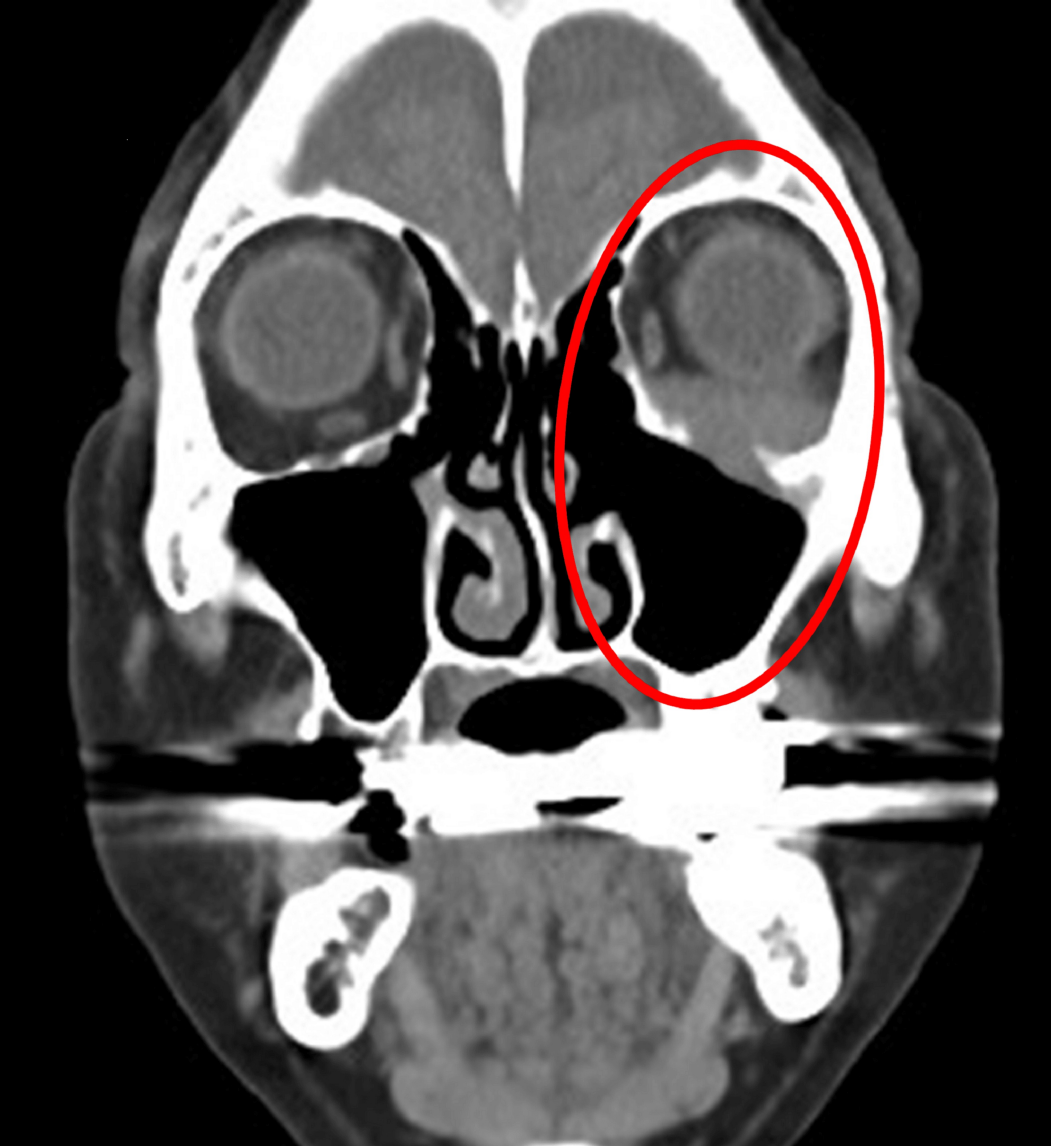
Plain CT image (after six months of treatment) Occupying lesion centered on the left maxillary sinus has improved (circle area). CT, computed tomography

The patient was started on oral prednisolone at a dose of 1 mg/kg/day (45 mg/day). His diplopia and orbital swelling improved significantly within three weeks. The corticosteroid dose was tapered gradually by 5 mg every three to five weeks, and then maintained at 5 mg per day. The patient remained symptom-free without recurrence for over 18 months (Figure 4).

## Discussion

RDD is a benign histiocytic proliferation with unclear etiology. Pathologically, emperipolesis - the presence of an intact cell within the cytoplasm of another S-100 protein-positive cell - is a particular finding in RDD [1]. Cervical lymphadenopathy without pain is observed in approximately 90% of cases, and fever, leukocytosis, hyperosmolarity, and hypergammaglobulinemia may also be observed [1,2]. In rare cases, RDD can affect the central nervous system, leading to neurological symptoms [3]. Extranodal lesions can occur in various regions, including the skin, nasal cavity, paranasal sinuses, orbit, bones, salivary glands, lungs, liver, kidneys, central nervous system, and upper respiratory tract. Of note, approximately 75% of extranodal lesions occur in the head and neck region [1,4]. Isolated paranasal sinus involvement is particularly uncommon and may mimic more aggressive conditions such as lymphoma, carcinoma, granulomatosis with polyangiitis, or IgG4-related diseases. In the present case, the absence of lymph node metastasis and normal laboratory results - such as sIL-2R and IgG4 levels (which tend to be elevated in the previously mentioned diseases) - made it challenging to make an accurate diagnosis for the lesion extending from the maxillary sinus to the orbit.

Diagnosis of RDD relies on histopathology. Emperipolesis - a hallmark feature - refers to intact lymphocytes or plasma cells engulfed by histiocytes. Immunohistochemistry aids in distinguishing RDD from other histiocytic disorders; RDD cells are typically positive for S-100 and CD68 and negative for CD1a, unlike Langerhans cell histiocytosis [4,5]. In the present case, the particular finding of RDD, emperipolesis, was not obtained on the initial histological examination, and the second examination led to the diagnosis of RDD. It is important to repeat the examination as needed.

Overall, the prognosis for RDD is generally good, and the course of the disease varies widely, including spontaneous resolution in some cases [6,7]. Therefore, treatment options depend on the severity and location of the disease, ranging from observation to interventions such as corticosteroids, chemotherapy, radiotherapy, or surgery. Observation may be appropriate for asymptomatic cases; however, in cases involving vital structures or causing functional impairment, systemic corticosteroids are commonly employed [5,8,9]. Some reports suggest that prednisone doses ranging from 40 to 70 mg/day may lead to partial or complete response [8,10]. In steroid-refractory or relapsed cases, immunosuppressants, chemotherapy, or surgical debulking may be considered [8]. However, other reports on orbital, tracheal, renal, or soft tissue RDD have described cases that did not respond to steroids [11]. There is also a risk of relapse after steroid discontinuation. In our case, due to the lesion’s proximity to the orbit and potential functional morbidity from surgery or radiotherapy, steroid therapy was selected initially. The patient responded favorably to corticosteroids, with no relapse following a gradual taper. It was useful to perform periodic CT scans for monitoring the lesions, as they were not easily visible from the surface.

## Conclusions

This case highlights the diagnostic challenge posed by RDD in the absence of lymphadenopathy and with radiographic features suggestive of malignancy. RDD should be included in the differential diagnosis of destructive maxillary sinus lesions, particularly when involving the orbit. Accurate diagnosis, through biopsy and immunohistochemistry, is critical to guide appropriate treatment. Corticosteroid therapy may offer effective symptom relief and long-term control in select cases.
